# Tripartite interactions: *Leishmania*, microbiota and *Lutzomyia longipalpis*

**DOI:** 10.1371/journal.pntd.0008666

**Published:** 2020-10-14

**Authors:** Thais Bonifácio Campolina, Luis Eduardo Martinez Villegas, Carolina Cunha Monteiro, Paulo Filemon Paolucci Pimenta, Nagila Francinete Costa Secundino

**Affiliations:** Laboratory of Medical Entomology, René Rachou Institute–FIOCRUZ, Minas Gerais, Brazil; The Faculty of Medicine, The Hebrew University of Jerusalem, ISRAEL

## Abstract

The microbial consortium associated with sandflies has gained relevance, with its composition shifting throughout distinct developmental stages, being strongly influenced by the surroundings and food sources. The bacterial components of the microbiota can interfere with *Leishmania* development inside the sandfly vector. Microbiota diversity and host-microbiota-pathogen interactions regarding New World sandfly species have yet to be thoroughly studied, particularly in *Lutzomyia longipalpis*, the primary vector of visceral leishmaniasis in Brazil.The native microbiota of different developmental stages and physiological conditions of *Lu*. *longipalpis* (Lapinha Cave), was described by culturing and 16s rRNA gene sequencing. The 16s rRNA sequencing of culture-dependent revealed 13 distinct bacterial genera (*Bacillus*, *Enterococcus*, *Erwinia*, *Enterobacter*, *Escherichia*, *Klebsiella*, *Lysinibacillus*, *Pseudocitrobacter*, *Providencia*, *Pseudomonas*, *Serratia*, *Staphylococcus* and *Solibacillus*). The *in vitro* and *in vivo* effects of each one of the 13 native bacteria from the *Lu*. *longipalpis* were analyzed by co-cultivation with promastigotes of *L*.*i*. *chagasi*, *L*. *major*, *L*. *amazonensis*, and *L*. *braziliensis*. After 24 h of co-cultivation, a growth reduction observed in all parasite species. When the parasites were co-cultivated with *Lysinibacillus*, all parasites of *L*. *infantum chagasi* and *L*. *amazonensis* died within 24 hours. In the *in vivo* co-infection of *L*.*chagasi*, *L*. *major* and *L*. *amazonensis* with the genera *Lysinibacillus*, *Pseudocitrobacter* and *Serratia* it was possible to observe a significant difference between the groups co-infected with the bacterial genera and the control group.These findings suggest that symbiont bacteria (*Lysinibacillus*, *Serratia*, and *Pseudocitrobacter*) are potential candidates for paratransgenic or biological control. Further studies are needed to identify the nature of the effector molecules involved in reducing the vector competence for *Leishmania*.

## Author summary

According to the World Health Organization Leishmaniasis is the second parasitic disease that kills the most in the world; the first is malaria. Despite this, knowledge about the *Leishmania* parasite and its interaction with vertebrate hosts concerning the transmitting insect is still relatively fewer and fragmented. Studies on insects microbiota have great importance to obtain basic information. How a vector responds to the presence of different microorganisms and how they interact with various pathogens and may lead to the development of new strategies or tools that can be used to prevent or hinder the transmission of the protozoan by the vector insect. Considering the knowledge about the intestinal microbiota of sandflies, we aim to study the effect of bacterial isolates on *Lu*. *longipalpis* infection by different species of *Leishmania*, and it believed that these bacteria might influence the development of *Leishmania*, preventing, and hindering transmission, contributing to Leishmaniasis control strategies.

## Introduction

In the last decade, microbial communities associated with sandflies gained relevance, as it has observed to play an essential role in *Leishmania* development within the host’s digestive tract [1]. The sandfly microbiota is a dynamic community mostly acquired from their environment as in other Insecta or Diptera. Its composition shifts throughout its distinct developmental stages, being strongly influenced by the surroundings and food sources crossed during their life cycle [2,3,4]. For example, sandflies acquire bacterial symbiont as immature stages by feeding upon the organic matter from the humid soil in which they develop. From this larval food, some symbionts remain prevalent as members of the adult’s microbiota, suggesting a transstadial transmission after the pupation process [2,3]. In addition to niche-acquired symbionts, feeding sources may modify adult microbiota diversity. Both females and males feed on plant sap and honeydew aphids.

However, only females feed on blood, which can acquire from a variety of vertebrate hosts, and which required for egg development [4]. Microorganisms like bacteria, fungi, and protozoan parasites ingested during blood feeding can become established within the autochthonous community and modulate insect vectorial capacity [4–7].

The ingested blood meal is digested and processed inside the midgut, where distinct parasites, like *Leishmania*, develop to be transmitted by the vector. However, after the infective blood-meal, the parasite has to survive multiple host barriers. These may impact a mature infection since it affects the resistance to digestive enzymes, colonization of midgut, parasite differentiation, including metacyclogenesis [8,9]. The host barriers involving interactions between the microbial communities and *Leishmania* during its life cycle within the insect remain unclear.

Some studies have demonstrated the microbiota bacterial components interfering with *Leishmania* development inside the sandfly vector. For instance, *Serratia marcescens*, a well known pathogenic bacteria for many insects [10], negatively interacted with *Leishmania infantum chagasi* and *Leishmania braziliensis*, inducing lysis of the parasite cell membrane [11,12]. An *in vivo* study revealed reduced infection rates of *Leishmania mexicana* in *Lutzomyia longipalpis* sandflies previously fed on a microbial suspension of *Pseudozyma sp*., *Asaia sp*. or *Ochrobactrum intermedium* [5]. The same study showed a pre-infected sandfly challenged with a high dose (5x10^7^ CFU/ml) of *S*. *marcensces* survived longer compared to control (uninfected but challenged with bacteria). These results suggest that *Leishmania* directly protects the sandfly from the bacterial infection, or modulates its effect by priming the host immune response, as observed in other models like *Anopheles gambiae* infected with *Plasmodium* [13]. Also, Hanssan et al. [3] showed that *Phlebotomus papatasi*, after antibiotic treatment became more susceptible to *Leishmania major* infection compared with the untreated control, suggesting that resistance to *Leishmania* infection was due to the presence of symbionts.

By contrast to the studies showing an adverse effect of the microbiota on *Leishmania* development, recent results suggest that the native microbiota is essential for *Leishmania* development and survival, with antibiotic treatment of sandflies inhibiting parasite growth and differentiation into the infectious metacyclic form [1,14]. In-depth knowledge of the underlying dynamics between bacteria and a host is needed to identify candidate bacteria that can be used in paratransgenic studies, or other biological approaches, aiming to control sandfly populations and *Leishmania* transmission [15].

In this context, the microbiota diversity and the host-microbiota-pathogen interactions regarding New World sandfly species have yet to be thoroughly studied, with only a few reported studies from Brazil, one from Argentina and one from Colombia [2,14,16–21].

In this report, we describe the culture-dependent native microbiota associated with *Lu*. *longipalpis*, a vector of visceral leishmaniasis. We assessed whether co-culturing members of associated microbiota and *Leishmania* would inhibit parasite growth rates *in vitro* and *in vivo*.

## Materials and methods

### Ethics statement

This study was conducted by the Oswaldo Cruz Foundation's Handbook for Animal Use (Ministry of Health of Brazil. It approved by The Animal Use Ethics Committee, Oswaldo Cruz Foundation (number LW-17/15).

### Sandflies

Wild-caught *Lu*. *longipalpis* were collected with CDC traps [22] at Lapinha Cave located in the city of Lagoa Santa, 60 km from Belo Horizonte (longitude 43°57’W; latitude 19°03’S), state of Minas Gerais, Brazil, a non-endemic region for transmission of Visceral Leishmaniasis. The adults collected with a CDC trap [22]. These traps placed in the early afternoon and removed in the following day morning, over four years during the spring to the summer season, and an average of 800 sandflies per collection. Immediately after the field trip, the insects collected kept at the insectary of the Laboratory of Medical Entomology of the René Rachou Institute (IRR) with constant temperature and humidity (25°C and 75% humidity). The flies were morphologically identified, according to Young and Duncan [23], and processed following midgut contents [16]. A sub-sample of *Lu*. *longipalpis* females scored as blood-fed were allowed to lay eggs. Larva from hatched eggs was maintained as described by Secundino & Pimenta, [24]. Immature stages (parental line), larvae, and pupae used for posterior analyses.

For *in vivo* experiments, flies were collected in the field separated under a scope, and the non-fed females maintained with a filtered 10% sucrose *ad libitum* at the insectary.

### Specimen preparation

Samples previously immobilized at -20 for few seconds and their surfaces sterilized by one-minute submersion in 1% hypochlorite, 15 to 30 seconds in 70% ethanol, and then rinsed three times with PBS [25]. After that, immature forms (larvae and pupae) and adults (midguts) with specific midgut contents: i) UF (fed on sucrose, non-blood-fed); ii) BF (Uninfected blood-fed); iii) GR (Gravid, empty midguts without blood and with developed ovaries), iv) P/S treated (sandflies pre-treated with Pen Strep for three days) and vi) PI (post coinfection with *L*.*i*. *chagasi* and bacterial isolates) identified and processed. Also, the internal controls added (insect carcass imprint, media alone). All the samples were pooled in groups of 15 (individuals) for culture-dependent bacterial profiling, in a minimum of 3 replicates each group.

### Bacterial diversity profiling using a culture-dependent technique

Bacterial members of the microbiota within each pool were isolated as follows: samples were homogenized following [26], in tubes containing 200 μL of brain-heart infusions broth (BHI) (Sigma, Missouri, United States), a non-selective medium, to promote the growth of an ample range of bacteria. The homogenates (100 μL) were then pour-plated in the BHI agar medium at 27°C for 48 hours. The observed colonies, differentiated by color and morphologic characteristics, were subjected to three passages in agar medium. The pure colonies expanded in the liquid medium, and each of the isolates differentiated by Gram staining and taxonomical profile. Finally, after the *in vitro* assays, the bacteria were re-plated and re-sequenced to ensure that the bacterial genera remained the same.

### 16S rRNA oriented profiling

The bacterial gDNA extraction was performed with DNeasy Blood & Tissue kit (Qiagen, Hilden, Germany) according to the manufacturer's instructions. The genomic material was quantified, and its purity assessed using the NanoDrop Spectrophotometer (Thermo Fisher Scientific). Bacterial gDNA from each sample served as a template for a PCR using Illustra PuReTaq Ready-To-Go PCR Beads (GE Healthcare, Buckinghamshire, United Kingdom) and primers 16S ribosomal RNA 27 sense 5'-AGAGTTTGATCA/CTGGCTCAG-3', and 1492 antisense 5'-TACGGT/CTACCTTGTTACGACTT-3'.

Amplification conditions were a 96 C° hold for 2 min, 30 cycles of 95°C for 1 min, 50°C for 1 min, and 72°C for 3 min, followed by 5 min on 72°C. Amplified products were visualized on a 1% agarose (Fisher Bioreagents, New Hampshire, United States) gel and cleaned using Wizard SV Gel and PCR Clean-up System (Promega, Wisconsin, United States). Twenty ng of the purified PCR product was sequenced using the above-described PCR primers and the DyeNamic ET Terminator on a DNA Sequencer—ABI 3730 (Life Technologies, California, United States). Sequences were aligned, merged into contigs, and trimmed using Sequencer software (version 5.4.6). Resulting sequences were analyzed searching for similarity, using the sequence analysis tool RDP (Ribosomal Database Project—Update 5) and against the NR database (Non-redundant, NCBI database) using BLASTN algorithm with default parameters [27]. The best BLAST hit was selected, considering a 97% identity threshold. Taxonomic profiling of bacterial communities using 16S rRNA sequences as a target has some well-known limitations, including database bias [e.g., due to a small amount of sandfly associated curated sequences deposited]. The differences in each 16S rRNA variable regions exhibit when resolving taxonomic levels [28,29], and even cross-kingdom amplification [30] amongst other issues [31,32]. We cautiously chose to report taxonomic identifications at the genus level only.

### Parasites

*L*. *i*. *chagasi* (MHOM/ BR/1970/BH46), *L*. *amazonensis* (IFLA/BR/67/PH8), *L*. *braziliensis* (MHOM/BR/75/M2903) and *L*. *major* (MHOM/IL/80/FN). Promastigotes were cultured in Medium 199 (Sigma, Missouri, United States) supplemented with 10% fetal bovine serum (Cultilab, São Paulo, Brazil), and other components: penicillin (100 U/ml) (Gibco, California, United States), streptomycin (50 mg/ml) (Gibco, California, United States), Hepes (40 mM) (Sigma, Missouri, United States), adenine (0.1 mM) (Sigma, Missouri, United States) and hemin (2.5 mg/ml) (Sigma, Missouri, United States) at 26°C [33].

### *In vitro Leishmania* co-culture

Parasites were washed twice in sterile Phosphate-buffered saline (PBS), counted, and re-suspended in fresh DMEM (Dulbecco's Modified Eagle Medium) (Sigma, Missouri, United States). Afterward, mixed with native bacteria previously isolated from sandflies, (as described below) and both (parasites and bacteria) were co-cultured in DMEM.

The number of bacteria was estimated following the colony-forming units (CFU) counting technique, using serial dilutions in Petri dish with BHI medium. The 10^−8^ dilution it was possible to count 300 CFU that used in all experiments. i) Isolate assays performed using 4x10^6^ parasites and 10^8^ bacteria / mL. Each parasite strain (listed above) and a single bacterial isolate (Tables 1 and 2) were incubated at 25°C, 0hr to 120 hours. ii) Supernatant assay, the bacterium culture was grown for 16 hours in the BHI medium; after that, centrifuged for 10 minutes at 15000xg, and only the supernatant (100μl) used for the co-culture experiments. The samples were co-cultured (parasites and supernatant) at 25°C through 0hr to 120 hours [11,12]. In the end, the number of alive promastigotes estimated by counting in a hemocytometer. After *in vitro* assays, the bacteria were re-plated and re-sequenced to ensure that the bacterial genera remained the same.

**Table 1:** Bacterial genera isolated from different developmental stages and physiological conditions of *Lu*. *Longipalpis*.

Genus /Gram	*Lu*. *longiplapis*				
	Larvae	Pupae	UF	BF	GR
***Bacillus* (+)**		X	X		
***Enterobacter* (-)**			X		
***Enterococcus* (+)**			X		
***Erwinia* (-)**			X		X
***Escherichia* (-)**			X		
***Lysinibacillus* (+)**	X		X		
***Providencia* (-)**			X		X
***Pseudomonas* (-)**	X	X			
***Serratia* (-)**				X	
***Staplylococcus* (+)**			X		

(+) Gram-positive and (-) Gram-negative; UF = Non-blood-fed; BF = Uninfected blood-fed; GR = Gravid.

**Table 2:** Bacterial genera isolated from *Lu*. *Longipalpis* after treatment with PenStrep and post-infection with *L*.*i*. *chagasi*.

Genus /Gram	*Lu*. *longiplapis*		
	UF	PS TREATED	PI
***Bacillus* (+)**	X		
***Enterobacter* (-)**	X	X	X
***Enterococcus* (+)**	X		
***Erwinia* (-)**	X		
***Escherichia* (-)**	X		X
***Klebsiella* (-)**		X	
***Lysinibacillus* (+)**	X		
***Providencia* (-)**	X		
***Pseudocitrobacter* (-)**		X	
***Serratia* (-)**			X
***Solibacillus* (+)**	X		
***Staplylococcus* (+)**	X		

(+) Gram-positive and (-) Gram-negative; UF = Non-blood-fed; PS TREATED = Sandflies pre-treated with Pen Strep; PI = Post infection.

### *In vivo*—sandfly infection

Groups of 150 flies previously treated with 50 U/ml penicillin plus 50μg/ml streptomycin (daily changed) in the sugar meal three days before the infective meal [1, 34].

After that, flies fed through a chick skin membrane apparatus filled with heparinized mouse blood reconstituted with heat-inactivated serum, plus 4 × 10^6^mL logarithmic phase promastigotes and bacterial isolates at 1 x 10^8^ bacteria/ mL.

As a control, one group fed only with blood and parasites, and another group only pre-treated with the antibiotic (Pen Strep) were analyzed. After the infective blood meal, engorged flies were separated and maintained at 27°C and 75% humidity and provided 10% sucrose *ad libitum*. The sandflies were analyzed three and six days post-infection (d.p.i).

Ten flies were frozen (-20°C) and killed in a 5% soap solution, and then midguts were dissected and transferred individually into a tube containing 30 μl PBS. The midguts, individually homogenized using a plastic pestle, then10μl of the supernatant (containing the released promastigotes) counted under a hemocytometer, each experiment was performed three times.

### Bacteria add back

The bacteria added after the experimental infection with *L*.*i*. *chagasi* carried out following the previously described protocol (*in vivo*–sandfly infection). Wild-caught sandflies previously treated for three days with Pen Strep and then experimentally infected with *L*.*i*.*chagasi* (4 × 10^6^ mL logarithmic phase promastigotes). Three days after the infective blood meal, at the end of the digestive process, a sterile solution of sucrose with 1x10^8^ bacteria/ mL of *Lysinibacillus* or *Serratia* was offered to the flies. The flies were analyzed three days after the addition of bacteria, corresponding to six d.p.i.

### Statistical analysis

The results were analyzed using GraphPad Prism 7. The non-parametric Kruskal-Wallis test (ANOVA) used for statistical analysis. Values of p < 0.05 were considered significant. *P<0.05, **P<0.001, ***P<0.0001 and ****P<0.00001.

## Results

### Sandfly native microbiota diversity revealed by a culture-dependent method

The 16s rRNA gene sequencing of cultivated bacteria from the native microbiota of *Lu*. *longipalpis* showed shifts in the community composition (in terms of genus richness) across the tested life stages, as well as in the distinct physiological conditions evaluated. A total of 92 CFUs of all groups were isolated and sequenced, revealing ten bacterial genera (*Bacillus*, *Enterococcus*, *Erwinia*, *Enterobacter*, *Escherichia*, *Lysinibacillus*, *Providencia*, *Pseudomonas*, *Serratia*, and *Staphylococcus*). General aspects, 54% had a whitish color, and all colonies were circular, the predominant elevation and margin type were umbonate and entire (56%), respectively; six were Gram-negatives (60%), and four were Gram-positives (40%) (Table 1).

### Immature forms

Regarding larval and pupal stages, there were respectively three genera (*Bacillus*, *Lysinibacillus*, and *Pseudomonas*) and two families (Bacillaceae and Pseudomonadaceae) present in the isolated colonies, with only *Pseudomonas* present in both groups.

### Adults

The UF group showed the highest bacterial diversity, with eight different genera. Being four Gram-positive (*Bacillus*, *Enterococcus*, *Lysinibacillus* and *Staphylococcus*), and four Gram-negative (*Erwinia*, *Enterobacter*, *Escherichia* and *Providencia)*. Except for *Lysinibacillus*, *Bacillus* (Bacillaceae), and *Staphylococcus* (Staphylococaceae), the other isolates were Enterobacteriaceae family members. The only genus identified associated with the BF group was *Serratia*, a Gram-negative Enterobacteriaceae. Two distinct genera identified in the GR group, (*Erwinia* and *Providencia*), both Gram-negative Enterobacteriaceae.

Finally, genera shared between immature and adults groups were *Bacillus* and *Lysinibacillus*, Gram-positive taxa, identified in pupae and UF, and larvae and UF group, respectively.

### *In vitro* assay: short and long evaluation

#### The effect of *Lu*. *longipalpis* native microbiota on the survival of *Leishmania* species

**Fig 1:**
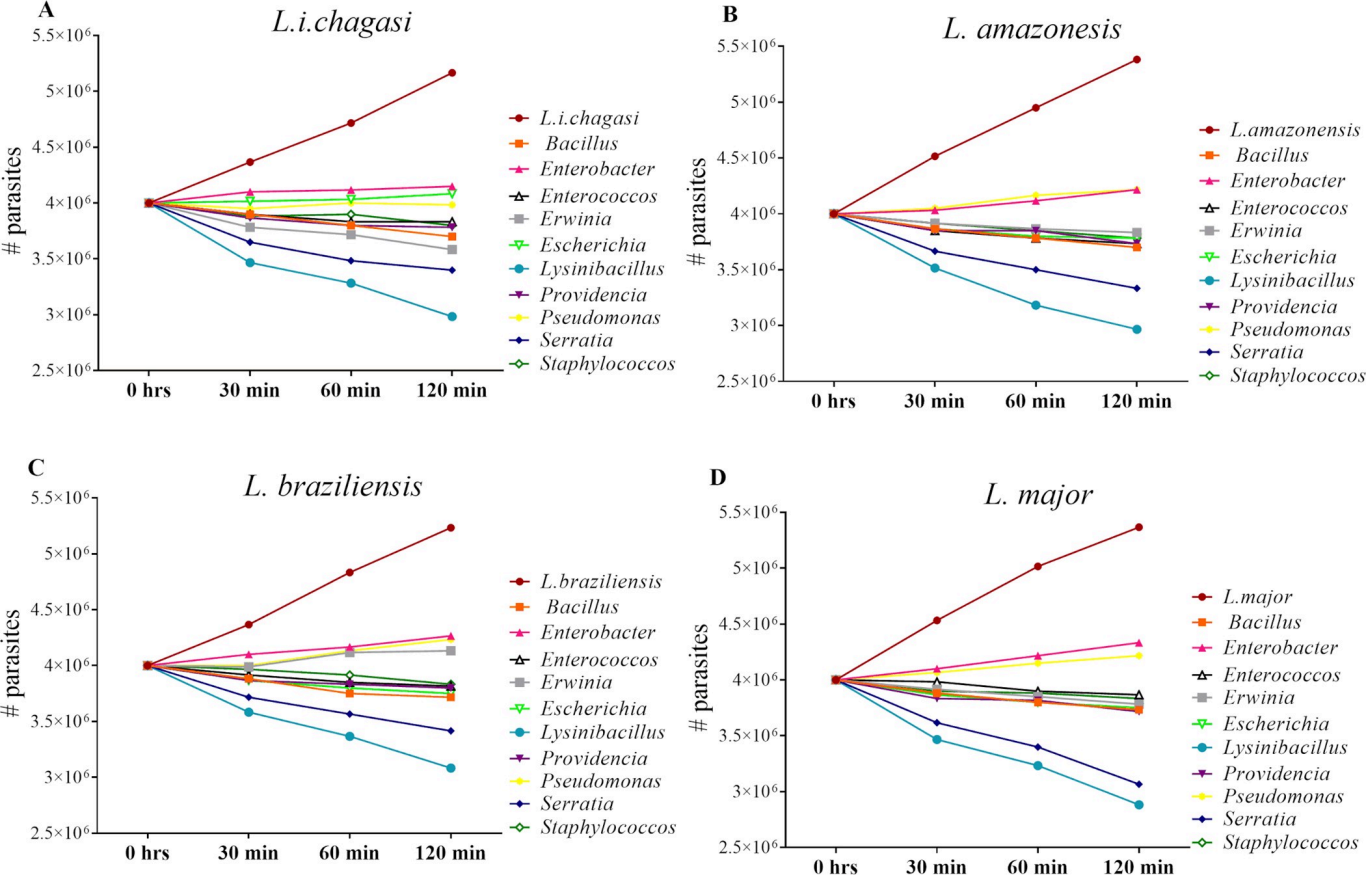
The short effect, in the parasite, when growth co-cultured with native bacteria. A short temporal evaluation (0 to 120 minutes) showing the activity of each one of the ten native bacteria isolated from *Lu*. *longipalpis* when co-culture with four *Leishmania* species: (A) *L*.*i*. *chagasi*, (B) *L*. *amazonensis*, (C) *L*. *braziliensis*, and (D) *L*. *major*.

i) Short time frame evaluation: The activity of each one of the ten native bacteria isolated from *Lu*. *longipalpis* were analyzed by co-cultivation with promastigotes of *L*.*i*. *chagasi*, *L*. *major*, *L*. *amazonensis*, and *L*. *braziliensis*. After 30 minutes of co-cultivation of the *Leishmania* promastigotes with the native bacteria, we observed a reduction in parasite numbers for all tested species. When compared to the 0 hr time point, the co-culture with *Lysinibacillus* and *Serratia* killed 13.25% and 8.75% of *L*. *chagasi*, respectively, and 12.25% and 8.5% of *L*. *amazonensis*, respectively. When these same bacteria were co-cultured with *L*. *brazilienesis* or *L*. *major*, they killed 10.5% and 7%, and 13.25% and 9.5%, respectively (Fig 1).

At 60 minutes, the killing persisted for the majority of co-cultures. At the 120 minutes time point, when comparing to the 0 hr mark, the respective killing of *L*. *chagasi*, *L*. *amazonensis*, *L*. *braziliensis* and *L*. *major* following co-culture with *Lysinibacillus* or *Serratia* was 25.5% & 15%, 26 & 16.75%, 23% & 14.5%, and 28% & 23.25%, respectively.

**Fig 2:**
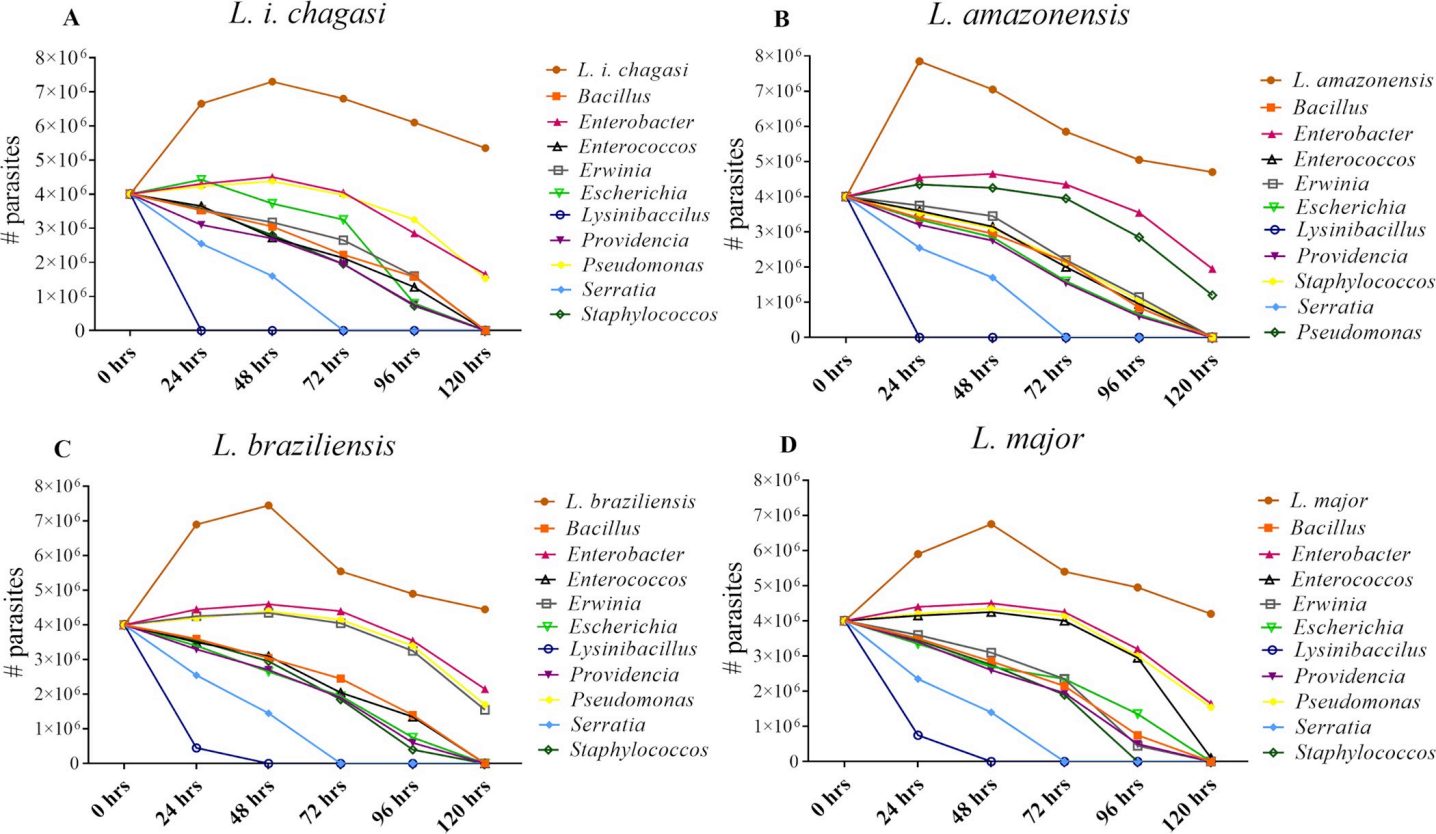
The long term mortality of *Leishmania* species when co-cultured with native bacteria. Over a 120 hrs. evaluation showing a reduction in parasite growth exerted upon four *Leishmania* species when co-cultured with ten bacterial genera isolated from *Lu*. *longipalpis*. (A) *L*.*i*. *chagasi*, (B) *L*. *amazonensis*, (C) *L*. *braziliensis*, and (D) *L*. *major*.

Statistically significant differences were observed for most groups when comparing parasite reduction numbers against the control (only the *Leishmania*) (α = 0.05). In particular, no significant reductions found when any parasite expose to *Enterobacter* and *Pseudomonas* and when *L*. *i*. *chagasi* (A) and *L*. *amazonensis* (B) were co-cultivated with *Escherichia* (Fig 1).

ii) Long time frame evaluation: After 24hrs of co-culture with associated sandfly bacteria, the *Leishmania* promastigote numbers decreased for all parasite species. Co-culture with *Serratia* killed 41.3% of *L*. *major*, and 36.25% of *L*.*i chagasi*, *or L*. *amazonensis* or *L*. *braziliensis* promastigotes (Fig 2). *Serratia* produced additional mortality in all species at 48 hrs, and after 72 hrs, all the parasites co-cultured with *Serratia* were dead. But, when the parasites were exposed to *Lysinibacillus* for 24 hrs, *L*.*i*. *chagasi* and *L*. *amazonensis* were totally killed, whereas the mortalities for *L*. *braziliensis* and *L*. *major* were 88.75% and 81.25%, respectively. After 48 hrs, all of the parasite species were completely dead (Fig 2).

At 72 hrs, all of the other co-cultured groups (*Bacillus*, *Enterobacter*, *Enterococcus*, *Erwinia*, *Escherichia*, *Staphylococcus*, *Providencia and Pseudomonas*) showed an inhibitory effect on bacterial growth or survival. Between 96 to 120 hrs, and in all co-cultured groups, the *Leishmania* parasites were failing or dead.

Statistically significant differences were detected for the majority of groups when comparing parasite reduction numbers between the control and the endpoints (α = 0.05). The exception was *L*. *major* with *Enterobacter* against the control group (only *Leishmania*) (Fig 2).

### *In vivo* assay

#### Evaluation of experimental coinfection *in vivo*—*Lu*. *longipalpis*, *L*. *i*. *chagasi* and bacterial isolates

**Fig 3:**
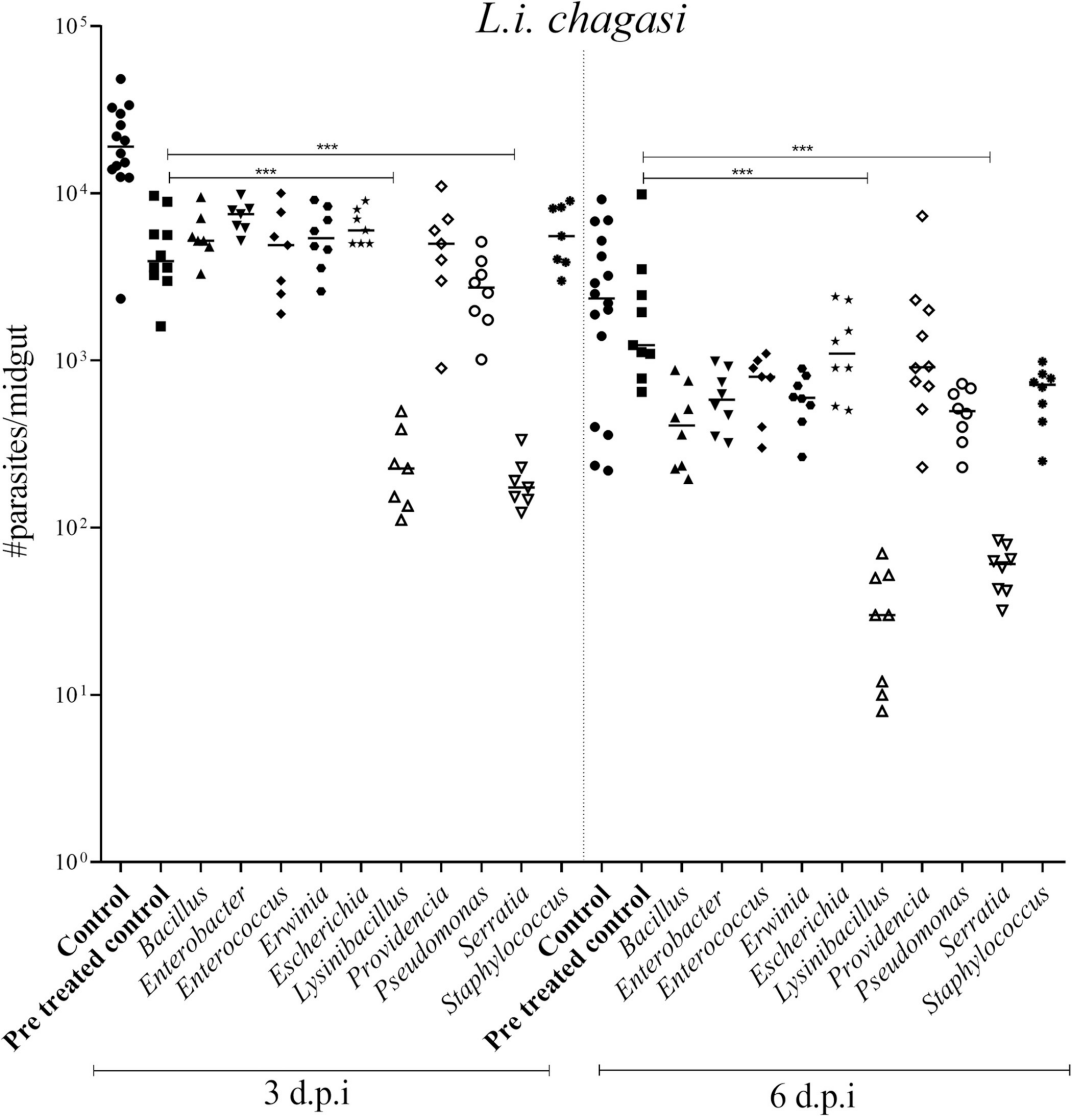
The effect of co-infection of *L*. *i*. *chagasi* and native bacteria. *In vivo* co-infection showing the result of the co-infection of *L*. *i*. *chagasi* (initial concentration 4x10^6^ parasites/mL) with all ten bacterial taxa (initial concentration of 10^8^ CFU/mL) isolated from *Lu*. *longipalpis*. The midguts individually macerated, and the number of parasites alive counted under a hemocytometer. The groups co-infected with *Serratia* and *Lysinibacillus* showed the lowest infection rate (statistically significant differences observed. α = 0.05). Six days post-infection, for all isolates, the number of parasites per midgut was lower than the pre-treated control.

Our *in vitro* results have shown that some bacterial genera have a powerful effect on inhibiting the survival of different *Leishmania* species, causing the death of promastigotes in a few hours or days. To test the effects of bacteria *in vivo*, all ten isolates obtained in this study were used to challenge *L*. *i*. *chagasi* infected *Lu*. *longipalpis* sandflies, a natural parasite—vector pair. Three days post-infective blood-meal, the number of parasites per midgut was detected, and the groups co-infected with *Serratia* and *Lysinibacillus* had 90% fewer parasites compared to the treated control group. At six d.p.i. for all isolates, the number of parasites per midgut was lower than controls. However, the co-infection with *Serratia* and *Lysinibacillus* again showed the lowest numbers of parasites per infected fly (Fig 3).

In summary the infection rate was 100% in all groups and the median of parasite per group was 7500 parasites/ per sandfly for *Bacillus* after 3 d.p.i and 408 parasites/ per sandfly after 6 d.p.i. (***). For *Enterobacter* the median was 5500 parasites/ per sandfly after 3 d.p.i (*) and 581 parasites/ per sandfly after 6 d.p.i. For *Enterococcus* the median was 4900 parasites/ per sandfly after 3 d.p.i. and 790 parasites/ per sandfly after 6 d.p.i. (*). For *Erwinia* the median was 5382 parasites/ per sandfly after 3 d.p.i and 592 parasites/ per sandfly after 6 d.p.i. (**). For *Escherichia* the median was 6000 parasites/ per sandfly after 3 d.p.i and 1100 parasites/ per sandfly after 6 d.p.i. For *Lysinibacillus* the median was 226 parasites/ per sandfly after 3 d.p.i (***) and 30 parasites/ per sandfly after 6 d.p.i. (****). For *Providencia* the median was 5000 parasites/ per sandfly after 3 d.p.i and 910 parasites/ per sandly after 6 d.p.i. For *Pseudomonas* the median was 2727 parasites/ per sandfly after 3 d.p.i and 497 parasites/ per sandfly after 6 d.p.i. (***). For *Serratia* the median was 174 parasites/ per sandfly after 3 d.p.i (***) and 58 parasites/ per sandfly after 6 d.p.i. (****). For *Staphylococcus* the median was 5545 parasites/ per sandfly after 3 d.p.i and 690 parasites/ per sandfly after 6 d.p.i. (**). And for the control group was 19020 parasites/ per sandfly after 3 d.p.i and 2201 parasites/ per sandfly after 6 d.p.i. For pretreated group the median was 3923 parasites/ per sandfly after 3 d.p.i and 1240 parasites/ per sandfly after 6 d.p.i.

### Composition of the bacterial community associated with *Lu*. *Longipalpis* after co-infection

The cultivable bacterial methodology showed the bacterial communities associated with *Lu*. *longipalpis* post-co infection composed by eight bacterial genera with the following composition: i) UF group—*Bacillus*, *Lysinibacillu*s and *Solibacillu*s, all Gram-positive; ii) P/S treated group—*Enterobacter*, *Klebsiella*, and *Pseudocitrobacter*, all Gram-negative; iii) PI group—*Enterobacte*r, *Escherichia*, and *Serratia*, all Gram-negative (Table 2).

Our results showed a shift in the composition of the bacterial community associated with *Lu*. *longipalpis* before and after infection (unfed and blood-fed), as follows: *Klebsiella*, *Pseudocitrobacter*, and *Solibacillus*. Also, those bacterial isolates effects were *in vitro* tested with *L*.*chagasi*, *L*.*amazonensis*, *L*.*braziliensis*, and *L*.*major* up to 120 hours (S1 Fig).

### Co-infection of *Lu*. *longipalpis* with native isolates of bacteria and *Leishmania* spp

After analysis of all the *in vitro* (Figs 1 and 2 and S1 Fig) and *in vivo* results (Fig 3), *Lysinibacillus*, *Pseudocitrobacter*, and *Serratia* showed to be the most competitive or aggressive bacteria against the parasites. These three isolates used for co-infection assays with *L*. *i*. *chagasi*, *L*. *amazonensis*, and *L*. *major*.

**Fig 4:**
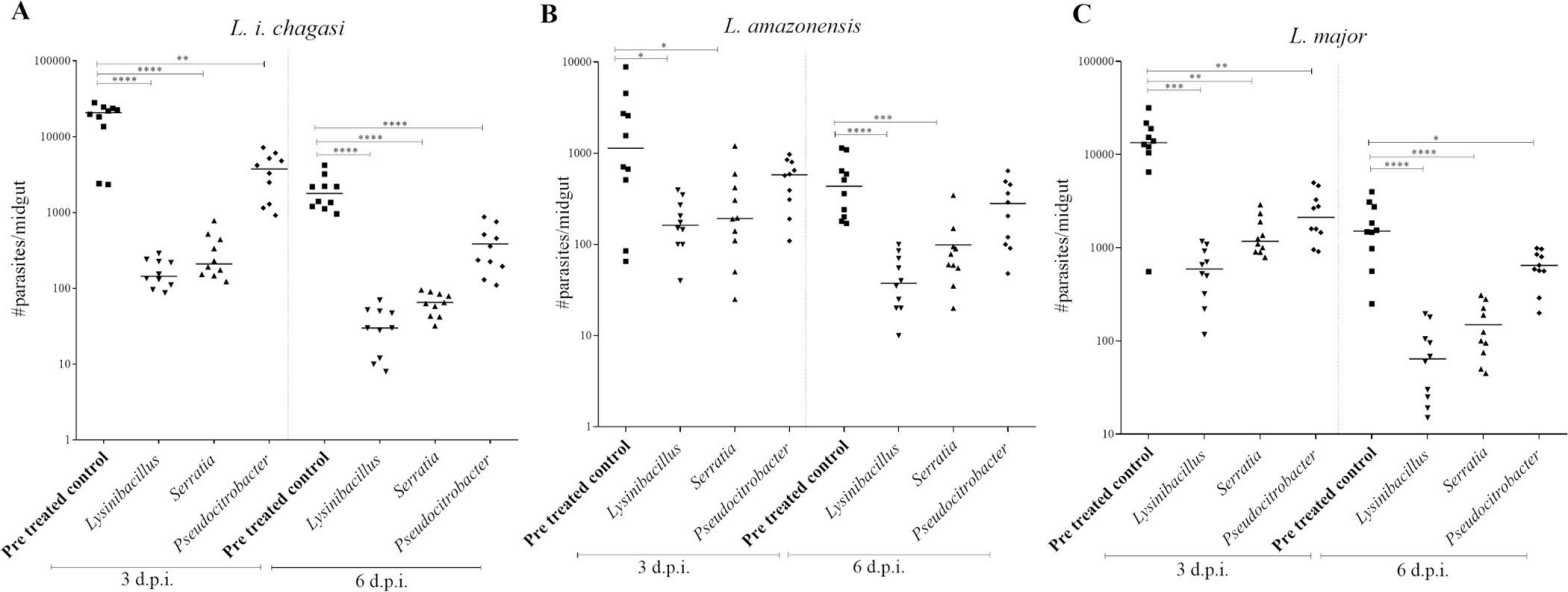
Co-infection with the native bacteria and the effect in the live parasite. *In vivo* co- infection of *L*. *i*. *chagasi* (A), *L*. *amazonensis* (B), and *L*. *major* (C) with the genera *Lysinibacillus*, *Pseudocitrobacter*, or *Serratia* affect live parasite counts. The sandflies were infected by artificial feeding with 4 × 10^6^mL logarithmic phase promastigotes and bacterial isolates at 1 x 10^8^ / mL. Three and six days post-infection the midguts were individually macerated Moreover, the number of parasites alive counted under a hemocytometer.

Again, a decrease in the number of parasites per midgut observed for all *Leishmania* species tested (Fig 4). Live parasite counts showed significant reductions in all the coinfected groups at both day three and six day d.p.i., and compared with the control group, similar to the *in vitro* results (Fig 4 and S1 Fig). The exception was *Pseudocitrobacter*, which was able to cause a significant decrease in the number of parasites in *L*. *i*. *chagasi* (median 396 parasites/per sandfly) and *L*. *major* (median 671 parasites/per sandfly), but not *L*. *amazonensis* (median 291 parasites/per sandfly).

In all the groups, co-infected tested had significant differences detected for bacterial effectors when compared with the control group, and the number of parasites per gut dropped similarly to the *in vitro* results (α = 0.05).

### Bacteria add back

To evaluate the effect of *Lysinibacillus* or *Serratia* after the establishment of *L*.*i*. *chagasi* infection in *Lu*. *longipalpis*, these two bacterial genera were offered to sandflies three days of post-blood infection. The experimental infection with *L*.*i*. *chagasi* carried out following the previous protocol. As a control, only infected sandflies, no bacterial genera offered. On the third day after bacterial addition, there was a significant decrease in the number of parasites per midgut for both *Lysinibacillus* (****) and *Serratia* (****) (S2 Fig).

## Discussion

In Díptera, the native microbiota plays many distinctive roles in the life cycle, such as development, immune response, reproductive biology, and vector competence. The intestinal diversity of microbiota associated with insect larvae and adults is variable and influenced by where they live and by their feeding behavior. Additionally, transstadial transmission is a mechanism that has been described in other arthropods [35].

In sandflies, the larval stages acquire different organisms because they feed in fertile soil where fungi and bacteria thrive [36]. Members of the microbiota acquired during the larval stage may remain autochthonously associated with the adults. Such a process implies that bacterial OTUs (Operational Taxonomic Units) would persist in the symbiotic assemblage throughout the whole insect metamorphosis [4, 37–39]. In anopheline mosquitoes, also observed that bacterial genera *Acinetobacter*, *Bacillus*, *Enterobacter*, *Staphylococcus*, *Pseudomonas*, *Cryseobacterium*, and *Serratia* persist as members of the insect microbiota throughout its life stages [40]. Here, we used the method of cultivable bacteria to address the native microbiota of *Lu*. *longipalpis* in immature stages and adults at distinct developmental stages and physiologic conditions.

A total of thirteen genera were identified, eight Gram-negative (61,5%), (*Erwinia*, *Enterobacter*, *Escherichia*, *Klebsiella*, *Pseudocitrobacter*, *Providencia*, *Pseudomonas* and *Serratia*) and five Gram-positive (38,5%) (*Bacillus*, *Enterococcus*, *Lysinibacillus*, *Staphylococcus*, and *Solibacillus*) with the majority identified as Enterobacteriaceae. These findings are in agreement with Oliveira et al. [20,21], who characterized the intestinal microbiota as prevalently Gram-negative in *Lu*. *longipalpis*, and with Kelly et al. [14], who defined Enterobacteriaceae as a dominant taxon under both sucrose-fed and blood-fed conditions.

Using a high throughput and NGS metagenomic analysis, [16,19,34], also reported the predominance of Gram-negative bacteria in blood feed and infected groups. The first study McCarthy et al. [19], analyzed males and females of *Lu*. *longipalpis* and Pires at al. [16] only females (from the same study location explored here), while the last study Monteiro et al. [34] targeted females of *Lu*. *intermedia* from a different geographical region and thus ecological niche.

In our studies, the genera *Bacillus* and *Lysinibacillus* were found in the immature stage (pupae) and adult, suggesting that species within these genera could remain transstadially associated to the sandfly. Volf et al. [4] showed that *P*. *dubosqui* featured a bacterial consortium associated with the gut immediately after adult emergence. Dillon et al. [6] had hinted the same in a study with *P*. *papatasi*, where the bacterial diversity profile within the sandfly gut was proposed to be dynamic, fluctuating during its lifespan. For example, in *Lutzomyia evansi* from field sites in Central America, the potential symbionts identified were *Enterobacter*, *Pseudomonas*, *Bacillus*, and *Lysobacter*, these taxa present across larvae, pupae and adults [41].

Regarding the immature stages, the genera we identified were *Bacillus*, *Lysinibacillus*, and *Pseudomonas*. In other studies involving laboratory colonized sandfly larvae were found, *Enterobacter* and Bacilli [1,42].

*Lysinibacillus*, Gram-positive, also founded in the adults (UF) along with *Staphylococcus* and *Solibacillus* (UF). Although less prevalent (38,5%), Gram-positive bacteria appear to be part of the shared microbiota of sandfly of the Old and New World. In the Old World, Maleki-Ravasan et al. [7,42] reported the Gram-positive *Bacillus*, *Staphylococcus*, and *Pseudomonas*. In contrast, Hillesland et al. [43] reported *Staphylococcus* and *Bacillus* in their studies with *Phlebotomus argentipes*, and the genus *Solibacillus* was described in *P*. *papatasi* [36]. In the New World, Oliveira et al. [20] and Pires et al. [16] found *Bacillus*, *Staphylococcus*, and *Pseudomonas* and Dey et al. [44] characterized the microbiota of *Lu*. *longipalpis* infected with *L*. *donovani*, identifying *Bacillus* and *Lysinibacillus*.

Also, the genus *Lysinibacillus* formally nominated as *Bacillus* spp. and well-known founded in soil and aquatic environments. Members of this genus can produce spores when submitted to adverse conditions [45]. A member of this taxon, *Lysinibacillus sphaericus*, named initially as *Bacillus sphaericus*, is used in commercial larvicides as part of effective insect control strategies. It has a well-documented toxic action on flies, especially for Culicidae [46–48].

In adult insects, bacterial isolates from the midgut, using a culture-dependent method, revealed predominantly bacteria belonging to the family Enterobacteriaceae (Gram-negative); *Erwinia*, *Enterobacter*, *Escherichia*, *Klebsiella*, *Serratia*, *Pseudocitrobacter* and *Providencia*. Enterobacteriaceae appears to be the most abundant family identified by other investigators [4,6,14,16,49,50]. That might be explained by the fast growth of these taxa in culture, which may outcompete the growth of other taxa [6].

Among the genera mentioned above, *Erwinia* and *Enterobacter* have been identified in plants [51,52]. Therefore, their presence could linked to adult feeding behavior on plant surfaces. Members of the *Escherichia* genus have also described in sandflies of the New and Old World [16,18,42,53,54]. *Providencia* is usually found in aquatic and terrestrial environments [55], is present in the hemolymph of *Drosophila melanogaster* [56] and the midgut of *Culex quinquefasciatus* [57] and *Lu*. *longipalpis* [16]. *Serratia* is a genera often identified in in sandflies [4,6,16–18,21,34,42,54,58]. *Pseudocitrobacter* and *Klebsiella* were associated with *Lu*. *longipalpis* post-infection. *Klebsiella* was already associated with *Lu*. *longipalpis* sandflies sampled from field sites (Alagoas, Brazil)[17].

During this study, we aimed to assess *in vitro* and *in vivo* if bacterial isolates from immature stages and different physiological states of *Lu*. *Longipalpis*, a permissive laboratory vector, had the potential to inhibit the growth and survival of *Leishmania* spp. In all co-cultures, *in vitro*, and *in vivo* tests, the presence of the metacyclic forms was observed a small number (1%). A possible explanation should be the decrease in pH caused by the presence of the bacteria, the medium composition, temperature, culture phase, and density of the parasite, and all these factors significantly influence the development of metacyclic promastigotes [59,60,61,62].

We reported an inhibitory effect of bacterial symbionts upon promastigotes of *L*. *infantum chagasi*, *L*. *major*, *L*. *amazonensis*, *and L*. *braziliensis*. In the *in vitro* co-cultures, *Lysinibacillus* and *Serratia* registered the most harmful effect for all species tested shows (Figs 1 and 2). Although *in vitro* studies showed a similar impact of co-cultured microbiota upon parasite survival has been reported for *Serratia*, *Bacillus*, and *Haemophilus parainfluenzae*, all of which induced lysis of promastigotes [11,12,3]. Also, we observe a reduction in the number of live parasites when the *Leishmania* spp. was co-cultivated with *Pseudocitrobacter* (S1 Fig).

A possible explanation for the parasite mortality is the formation of a structured bacterial community recognized as biofilms [63–64, 65]. Moraes et al. [11,12] observed by scanning electron microscopy that the bacteria *S*. *marcescens* (strain SM365) when incubated with *L*. *chagasi* and *L*. *braziliensis*, adhered to the entire cell body and flagellum of the parasite. Filamentous structures identified as biofilms formed, which could have induced the lysis of the cell membrane of the parasites. We do not rule out the hypothesis that other probable explanations may be due to the saturation of nutrients present in the culture medium, which leads to a faster death of the parasites due to the high multiplication of bacteria.

Several studies have already demonstrated the interference of bacteria upon insect infection by human pathogens [35]. It accepted that naturally occurring microorganisms in the midgut of insects might play an essential modulating role in the development of pathogens within their arthropod vectors.

In our *in vivo* studies, we tested all 13 isolates. It was showing that the genera *Lysinibacillus*, *Serratia*, and *Pseudocitrobacter* caused the death of the parasites within the midgut of the insect vector, the same as observed in the *in vitro* experiments. An *in vivo* study by Sant’Anna et al. [5] assessed how re-colonizing the intestines of females of *Lu*. *longipalpis* (Jacobina, Bahia) with bacteria and yeasts extracted from their midgut interfered in the capacity of the *L*.*mexicana* to colonize the insect host. A reduction was observed in the number of infected sandflies that had been pre-fed with *Pseudozyma sp*., *Asaia sp*. and *Ochrobactrum intermedium*, as well as a reduction in parasitic load.

In the study of Louradour et al. [1], the addition of *Enterobacter* and *Serratia* genera to the infective blood-meal promoted significant recovery of the number of metacyclic that developed in *P*. *duboscqi* treated with Pen Strep post-blood-meal excretion. Here, when both *Lysinibacillus* and *Serratia* were added after the establishment of the infection, a significant decrease in parasites number occurred (S2 Fig).

In summary, we showed that *Lysinibacillus*, *Pseudocitrobacter*, and *Serratia* strongly inhibited *Leishmania* growth and survival *in vitro* and co-infected *Lu*. *longipalpis*, the primary vector of American visceral leishmaniasis. After 24 h of *in vitro* co-cultivation of the *Leishmania* with the native bacteria, it was possible to observed growth reduction in all parasite species. When the parasites were co-cultivated with the bacterial genus *Lysinibacillus*, all parasites of *L*. *infantum chagasi* and *L*. *amazonensis* died within 24 hours. In the *in vivo* co-infection of *L*.*chagasi*, *L*. *major*, and *L*. *amazonensis* with the genera *Lysinibacillus*, *Pseudocitrobacter* and *Serratia*, it was possible to observe a significant difference between the groups co-infected with this bacterial genus and the control group pre-treated. These findings suggest that these three symbiont bacteria are potential candidates for paratransgenic or biological control. Further studies are needed to identify the nature of the effector molecules involved in reducing the vector competence for *Leishmania*.

## Supporting information

S1 Fig *Leishmania* species co-cultivated with bacteria isolated after an infective blood meal. Mortality observed over 120 hours with the four *Leishmania* species: (A) *L*.*i*. *chagasi*, (B) *L*. *amazonensis*, (C) *L*. *braziliensis*, and (D) *L*. *major*; co-cultivated with three distinct genera of bacteria (*Pseudocitrobacter*, *Klebsiella*, and *Solibacillus*) isolated from the midgut of *Lu*. *longipalpis* after infection blood meal with *L*.*i*. *chagasi*.
(TIFF)

S2 Fig *In vivo* assay showing the effect of addition bacteria after infection of the sandfly. To evaluate the effect of *Lysinibacillus* or *Serratia* after the establishment of *L*.*i*. *chagasi* (initial concentration 4x10^6^ parasites/mL) infection in *Lu*.*longipalpis*, these two bacterial genera were offered to sandflies (pre-treated with pen-strep) three days post the infectious (3 d.p.i) blood infection. Were provided a sterile solution of sucrose with 1x10^8^ CFU / mL of *Lysinibacillus* or *Serratia* and the flies were analyses three days after the bacterial feeding. It observed that on the third day after bacterial addition, there was a significant decrease in the number of parasites per midgut for both *Lysinibacillus* and *Serratia*. Statistical significance at α = 0.05.
(TIFF)
