# Supplementary figures and images for: Tripartite interactions: Leishmania, microbiota and Lutzomyia longipalpis
Source: PLoS Negl Trop Dis. 2020 Oct 14;14(10):e0008666. doi: 10.1371/journal.pntd.0008666 (PMC7556539; doi:10.1371/journal.pntd.0008666)

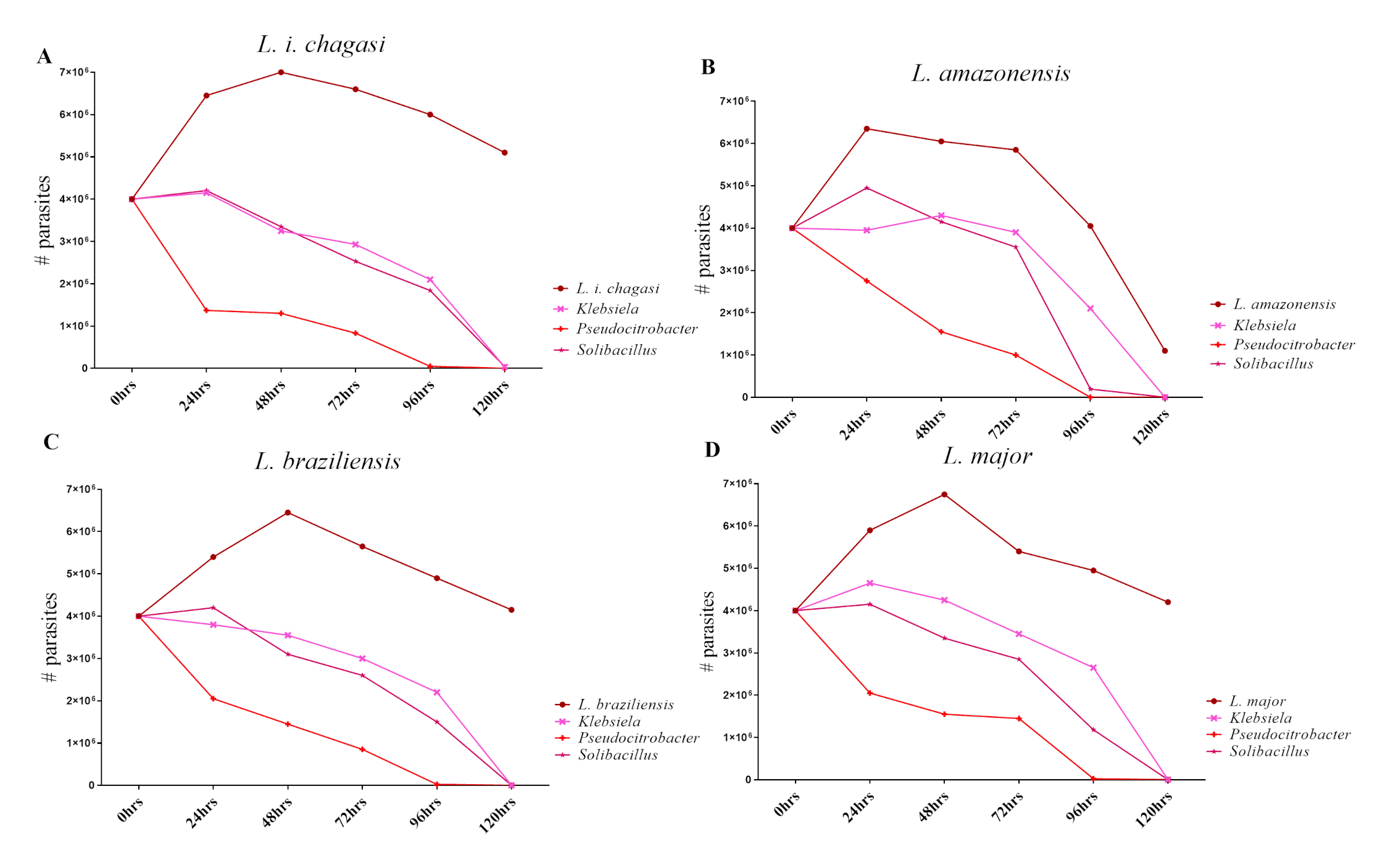

Supplement: S1 Fig — Mortality observed over 120 hours with the four Leishmania species: (A) L.i. chagasi, (B) L. amazonensis, (C) L. braziliensis, and (D) L. major; co-cultivated with three distinct genera of bacteria (Pseudocitrobacter, Klebsiella, and Solibacillus) isolated from the midgut of Lu. longipalpis after infection blood meal with L.i. chagasi. (TIFF) [file pntd.0008666.s001.tiff]

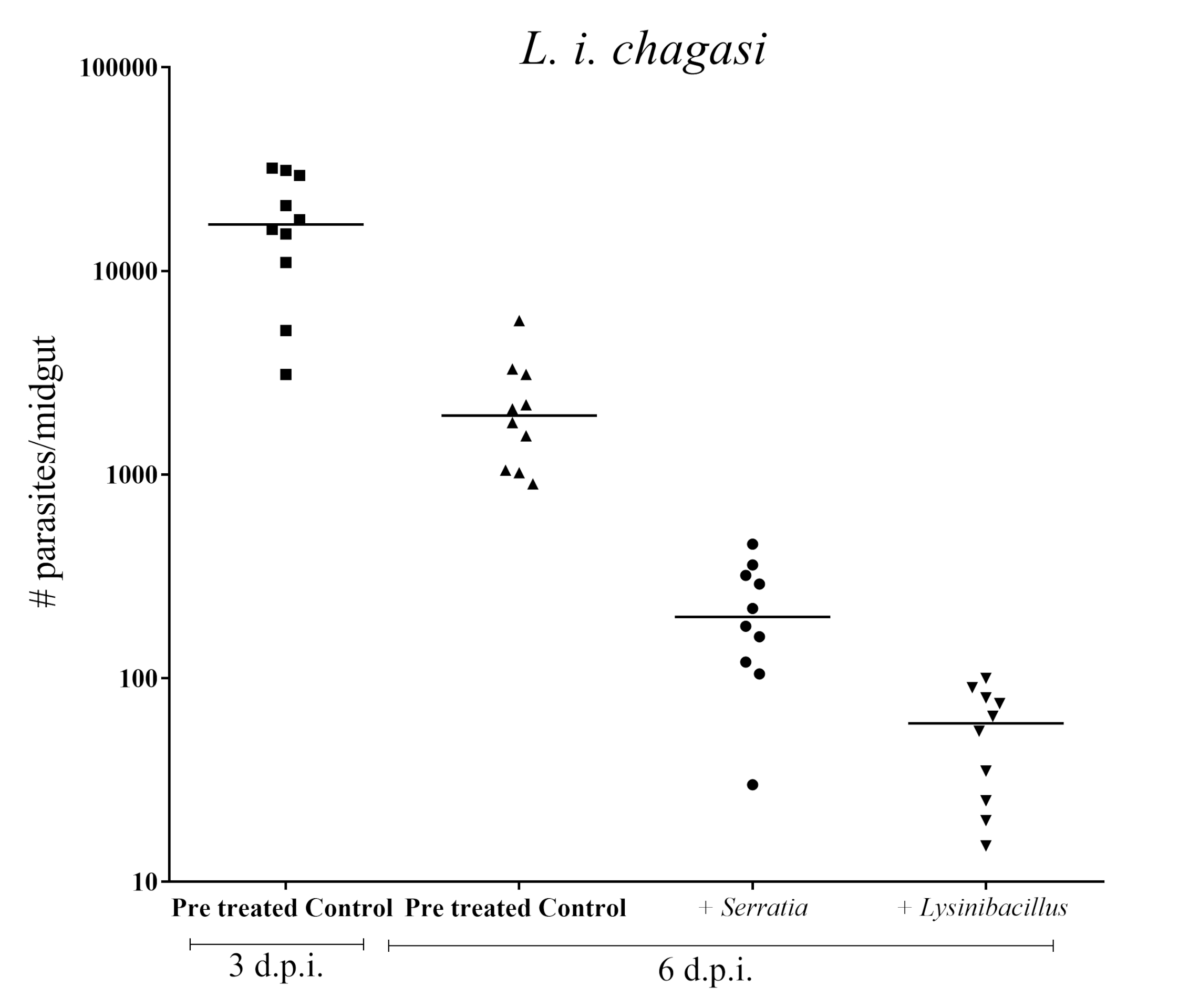

Supplement: S2 Fig — To evaluate the effect of Lysinibacillus or Serratia after the establishment of L.i. chagasi (initial concentration 4x10^6^ parasites/mL) infection in Lu.longipalpis, these two bacterial genera were offered to sandflies (pre-treated with pen-strep) three days post the infectious (3 d.p.i) blood infection. Were provided a sterile solution of sucrose with 1x10^8^ CFU / mL of Lysinibacillus or Serratia and the flies were analyses three days after the bacterial feeding. It observed that on the third day after bacterial addition, there was a significant decrease in the number of parasites per midgut for both Lysinibacillus and Serratia. Statistical significance at α = 0.05. (TIFF) [file pntd.0008666.s002.tiff]
